# Discussion in the Academy of Medicine of Paris, on Induction of Premature Labor

**Published:** 1852-10

**Authors:** 


*Discussion in the Academy of Medicine of Paris, on Induction of Premature Labor.*—A very animated debate has just been concluded in the Academy of Medicine of Paris, regarding induction of Premature Labor, in cases of extreme narrowing of the pelvis and of uncontrollable vomiting. The principal speakers were M. Cazeaux, the reporter of a paper on the subject, and M. P. Dubois. These gentlemen, who both hold an eminent position as obstetricians, disagree thoroughly on the question. M. Dubois advocates induction of premature labor in cases of obstinate vomiting, and rejects it in narrowing of the pelvis; whilst M. Cazeaux holds exactly contrary opinions. The paper which gave rise to the discussion was sent in by M. Lenoir, who requested the learned body to give an opinion on the question. He had, in fact, performed the operation upon a deformed woman, whose first child had been extracted in fragments, and he was anxious that the Academy should give its sanction to the proceeding, which sanction M. Dubois thought his colleagues should withhold.

We cannot enter into all the merits of the discussion, which was very animated and extremely instructive; we may, however, notice, that M. Dubois was proved to have himself advocated induction of premature labor in his lectures, and it was shown that his opinions must have since been materially modified. A feature of importance in the latter gentleman's speech is the relation of seven or eight cases of obstinate vomiting, which were mostly followed by death. In one of these cases induction of premature labor saved the patient and the child. M. Cazeaux showed very forcibly how preferable is induction of premature labor to the Cæsarian section or craniotomy; and the Academy, after a very protracted discussion, at last adopted the following conclusions, which are by far more reserved than those at first proposed by the reporter: "Whereas M. Lenoir was sufficiently warranted in having recourse to induction of premature labor in the case of Julie Gros, by the authority of two cases previously thus treated, and by the consenting voice of several of his professional brethren called in consultation, the Academy thanks the author for his interesting paper, and recommends the latter to the Committee entrusted with the publication of memoirs."—*London Lancet, Sept.* 2, 1852.

